# Do knowledge brokers facilitate implementation of the stroke guideline in clinical practice?

**DOI:** 10.1186/1472-6963-13-434

**Published:** 2013-10-23

**Authors:** Mia Willems, Carin Schröder, Marcel Post, Trudy van der Weijden, Anne Visser-Meily

**Affiliations:** 1 Rudolf Magnus Institute of Neuroscience and Center of Excellence for Rehabilitation Medicine, University Medical Center Utrecht and De Hoogstraat, Rembrandtkade 10, 3583, TM Utrecht Netherlands; 2 Department of General Practice, School Caphri, Maastricht University, Peter Debyeplein 1, 6229, HA Maastricht Netherlands

**Keywords:** Knowledge broker, Stroke, Guidelines, Implementation science, Theory of Planned Behavior

## Abstract

**Background:**

The implementation of clinical practice guidelines in rehabilitation practice is often troublesome and incomplete. An intervention to enhance the implementation of guidelines is the knowledge transfer program built around the activities of a knowledge broker (KB).

This study investigates the use of KBs to implement guideline recommendations for intensive therapy and physical activity for patients post-stroke in 22 stroke units in hospitals and rehabilitation centers in The Netherlands.

**Methods/Design:**

This study includes a quantitative evaluation with a non controlled pre-post intervention design and a mixed methods process evaluation. From each stroke unit, enterprising nurses and therapists will be recruited and trained as KB. The KB will work for one year on the implementation of the guideline recommendations in their team. To evaluate the effectiveness of the KB, a questionnaire will be administered to patients, health professionals and KBs at baseline (T0) and after one year (T1). Furthermore, semi structured interviews with 5 KBs will be performed at T1.

The primary outcome of this implementation project will be the support health professionals give patients to exercise and be physically active, as reported by patients and health professionals themselves. The support immediately after the intervention is compared with the support at the start of the intervention.

Additionally we will explore the influence of socio-demographic characteristics of health professionals and determinants identified in the Theory of Planned Behavior (intention, attitude, subjective norm and perceived behavioral control) on the change of supportive behavior of health professionals. Finally, KBs will complete a questionnaire on their own psychological and social demographic characteristics and on organizational conditions needed for health-care improvement such as time, workforce, sponsoring and support from management.

**Discussion:**

With this study we will gain insight in when and why knowledge brokers seem to be effective. Also we will identify determinants that predict which health professionals are susceptible to change their behavior. This study will provide guidance how to implement guidelines and will help to improve stroke rehabilitation services.

## Background

Stroke is one of the most common causes of death and acquired adult disability [1]. Although impressive developments have been made in the medical management of stroke, most post-stroke care relies on multidisciplinary rehabilitation interventions [2]. To describe appropriate care based on the best available scientific evidence clinical practice guidelines are designed. In 2009 in The Netherlands an updated clinical practice guideline for diagnosis and treatment of stroke was released. This guideline states that mobilization within 24 hours (level 2 evidence), 20–30 minutes of exercising twice a day under supervision of a therapist (level 1 evidence) and physical activity (level 3 evidence) lead to faster recovery in the first months post stroke [3]. The importance of exercise and physical activity was already acknowledged in the earlier clinical practice guideline from 2000 [4], but the recommendations in the current guideline are more specific.

Recent studies suggest that there is a considerable gap between these guideline recommendations and current practice. Hospitalized stroke-patients are inactive and spend most of the time alone in their room, in the Netherlands and in other countries [5,6]. This implies that these guideline recommendations have not been adequately implemented in medical practice to date.

It is a well-known problem that despite widespread dissemination and publicity, guidelines for medical practice are often not implemented effectively. There is a substantial gap between evidence and practice, with the result that best health outcomes are not achieved [7]. Many attempts have been made to improve the implementation of guidelines. Evaluations of these different interventions to improve implementation report modest effects but fail to produce a clear pattern of results favoring a particular strategy to draw on in developing effective interventions for knowledge transfer. One hypothesis, although this is not consistently found, is that multifaceted interventions are more likely to be effective than single interventions [8-10]. Furthermore there is evidence that interventions for implementation tailored to potential barriers to change are more effective than non-tailored interventions [11].

A multifaceted and tailored intervention that has gained interest for the implementation of guidelines is the knowledge transfer program built around the activities of a knowledge broker (KB). A KB is a research intermediary who helps to bridge the research-to-practice gap [12]. The specific activities of a KB are not standardized because the KB should be responsive to the needs of the stakeholders [13]. An effective KB has the ability to tailor key messages from research evidence to the local perspective [14], and thus incorporate best practices into existing routines [12].

Although there is growing interest in KBs, there is little evidence regarding the effect of KBs on professional practice or on patient outcomes [14,15]. Hardly anything is known about the most optimal organizational context, how to prepare and train a KB, and what personal characteristics determine KB effectiveness [14]. More knowledge of the determinants of success of KBs is therefore needed to design effective knowledge transfer programs.

**Figure 1:**
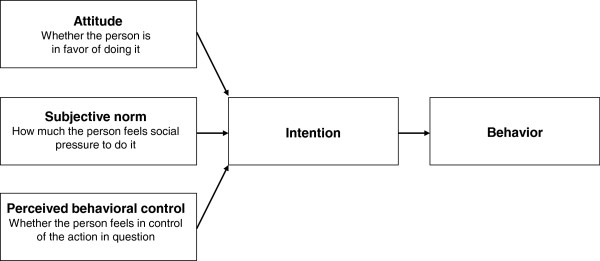
Theory of Planned Behavior.

Also, a better understanding is needed of why health-care professionals do or do not adopt guidelines. An important factor in the adoption of guidelines, is the individual decision of health professionals to change their behavior. The behavioral sciences provide a number of well-developed, operationalized, and tested models of human behavior. These models have demonstrated their value, also for the understanding and predicting intention and clinical behavior [16-18]. The Theory of Planned Behavior (TPB) is among the models with the best predictive utility. Figure 1 explains the theoretical framework of the TPB [19].

In this paper the protocol is described of a pre-post study aiming to evaluate the implementation of the clinical practice guideline recommendations to intensify therapy and physical activity in hospitals and rehabilitation centers in the Netherlands. Additionally, we will analyze determinants of behavior as described in the TPB. This allows us to improve our knowledge about the ability of KB to affect the behavior of the health professionals and/or the determinants of this behavior like intention, attitude, subjective norm and perceived behavior control.

The research questions are:

1. Does the support that health professionals give stroke patients to exercise and be physically active as reported by patients increase after the KB intervention? (patients perception)

2. Does the support that health professionals give stroke patients to exercise and be physically active as reported by the health professionals themselves increase after the KB intervention? (self reported supportive behavior of health professionals)

3. Are the determinants of behavior described by the Theory of Planned Behavior and social demographic characteristics of health professionals associated with the change in self reported supportive behavior of health professionals?

4. Is the change (self-reported and patient-reported) in supportive behavior of health professionals associated with social demographic and psychological characteristics of KBs and organizational context variables?

## Methods/Design

### Design of the study

**Figure 2:**
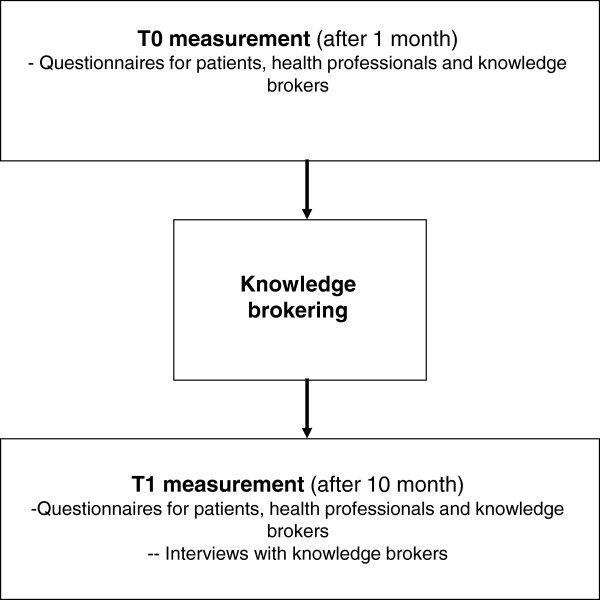
Study design.

This study includes a quantitative evaluation with a non controlled pre-post intervention design and a mixed methods process evaluation. The study design is presented in Figure 2. The intervention period will be one year. Questionnaires are administered at baseline (T0) and immediately after the intervention (T1) at one year. Furthermore, a purposeful sample of five KBs will be invited to participate in a semi structured interview at T1.

### Participants

Stroke units in hospitals and rehabilitation centers will be invited to take part in this study. To be included they will have to collaborate with a stroke unit in a hospital and write a motivation for participation. Furthermore for each unit an administrative director will have to sign an agreement to participate.

Members of the multidisciplinary stroke units, mainly nurses, physiotherapists and occupational therapists will all be asked to fill out a questionnaire before and after the KB intervention. We estimate there will be about 20 members per stroke unit.

Furthermore 15 inpatients per stroke unit will be interviewed before and after the KB intervention. This number is based on the number of stroke patients generally admitted to an average department of neurology. To be included in the interviews patients should be hospitalized, mobilized and able to answer questions.

### Intervention

#### Knowledge brokering

In each stroke unit two or three knowledge brokers will be appointed. Stroke units selects their own KBs. This are enterprising nurses, physiotherapists or occupational therapists. One KB has to be a nurse by profession, because nurses have a high potential to support and guide patients to be physically active as they are present during the whole day.

Goal of the KB intervention is to facilitate the use of the recommendations from the clinical practice guideline to intensify physical therapy and physical activity in the first weeks post stroke [3]. The specific activities of the KB will not be standardized because the role should be flexible and responsive to the needs of the stakeholders [13]. To support the KBs and their organizations we will employ the following activities:

1. Development of a KB job description. Qualifications sought for in the KB include expertise in neuro-rehabilitation, minimal 2 years of working-experience, persuasive personality, positive attitude to change, target driven and a team player.

2. Salary support for the KBs for two hours per week for one year.

3. Providing the KB with a number of existing “good practices” that can be used to intensify therapy and enhance physical activity (see paragraph materials). Furthermore the KB will receive a description of the behavior expected from health professionals to sufficiently support patient to exercise and be physically active (i.e. targeted behavior, see paragraph materials).

4. In the first two months of their appointment KBs will write a project plan that describes how they will implement the guideline-recommendations in their stroke unit. The KBs will be instructed to include in their plan the implementation of one “good practice” in their clinical practice and ways to stimulate their colleagues to sufficiently support patients to exercise and be physically active. Furthermore an obligatory activity will be to introduce themselves to their colleagues, assess barriers and supports to change and do an evaluation at the beginning and end of the implementation period. The national project coordinators (a physiotherapist and a project manager) will comment on the project plan.

5. Approximately every 2 months a face-to-face meeting will be organized for all KBs (8 meetings in total). During the first 4 meetings the focus will be on training the KBs in project-management skills, knowledge of neurorehabilitation and implementation strategies such as assessing barriers and supports to change, monitoring and evaluating outcomes en securing results. In the latter 4 meetings the focus will be on exchanging knowledge and experience amongst the KBs and with experts such as researchers, people with expertise on project management and patients.

6. In between the meetings KBs will be able to approach the national project coordinators with questions. The project coordinators will further encourage linkages between KBs, and with experts in neurorehabilitation. Collaboration amongst KBs will also be encouraged through a digital forum.

### Materials

#### Targeted behavior

To adhere to the recommendations that are subject of this study a specific supportive behavior is expected from health professionals (targeted behavior). This behavior has been described carefully and has been commented on by a broad group of experts in rehabilitation and adjusted according to their comments. Health professionals should for example stimulate patients to do as much as possible by themselves, give patients exercises to do by themselves and involve family in exercising.

#### Good practices

A broad call has been send to hospitals and rehabilitation centers to collect perceived best practices to intensify therapy and enhance physical activity. From these experiences experts in neurorehabilitation have selected a number of good practices. Good practices are evidence based, easy and efficient to implement. Examples of good practices are the use of a ‘do it yourself’ exercise-guide for stroke patients [20] and training of functional tasks during morning activities. For each good practice a fact-sheet has been drafted and scientific publications have been collected.

### Variables and measurements

Table 1 gives an overview of all variables that will be measured. Dutch versions of the questionnaires can be obtained from the first author.

**Table 1:** Overview of measures

	**Variables**	**T0**	**T1**
**Patients**			
Perceived support to be active (=adherence to the guideline)	Stimulate physical activity, functional tasks, instruct family	X	X
Institution	Hospital or rehabilitation center	X	X
Social demographic characteristics	Sex, age	X	X
Stroke characteristics	Type of stroke, hemisphere, date of onset, functional level (Barthel index [21])	X	X
**Health professionals**			
***Non-knowledge brokers***			
Support given to patients to be active (=Adherence to the guideline)	Behavior	X	X
Social demographic characteristics	Sex, profession, date of birth, working-experience	X	X
Determinants of Theory of Planned Behavior	Intention, attitude, subjective norm, perceived behavioral control	X	X
***Knowledge brokers***			
Social demographic characteristics	Sex, profession, date of birth, working-experience, education in neuro rehabilitation		X
Psychological characteristics	motivation, self efficacy (GSES-12 [22])		X
Result achieved	Goal achieved, satisfied with result, more intensive therapy		X
Organizational context	support of management, time, workforce, sponsoring		X

#### Outcome

##### Patients’ perception of support to be active

The support patients perceive from health professionals to exercise will be measured with 4 questions with a 4 point scale from not at all to completely. Patients will report if they feel stimulated to be physically active, if the feel stimulated by nurses to do functional tasks, if they feel stimulated by therapists to do functional tasks and if family received instruction about exercising. This questionnaire was piloted by three patients residing in a rehabilitation center.

### Supportive behavior of health-professionals

Supportive behavior of health-professionals will be measured by asking them to imagine a ordinary workday in the past two weeks and register for the patients they saw that day how many they supported to exercise and be physically active. Eight questions about the way support can be given to patients are formulated based on the targeted behavior (see materials). We ask for example how many patients they stimulated that day to do as much as possible by themselves and how often they involved family in exercising. To encourage health professionals to be realistic it will be emphasized that it is not possible to support each patients every day in all the different ways described.

#### Determinants that moderate change of health professionals

##### Determinants of Theory of Planned Behavior (Figure 1)

The 4 determinants of behavior described in the Theory of Planned Behavior (intention, attitude, subjective norm and perceived behavioral control) will be assessed to determine if they moderate change in the supportive behavior reported by health professionals. They will be assessed with a questionnaire developed following the manual for health service researchers ‘*Constructing questionnaires based on the Theory of Planned Behaviour’* using only direct measurements [19]. The questionnaire was piloted by five rehabilitation professionals working in stroke units not participating in our study and adjusted according to their comments.

*Behavior intention* will be assessed with three items measuring generalized intention to perform the taregeted supportive behavior. We will ask if they ‘expect to’ , ‘intend to’ and ‘want to’ perform the targeted behavior. These three items are measured on scales from 1 to 7 anchored by strongly disagree…strongly agree. Additionally an intention performance item will be used. Response is measured on a scale from 1 to 10.

The measure of *attitude* consists of six items using bipolar adjectives with a seven point response format. For example “good practice…bad practice” and “effective…not effective”.

The measure of *subjective norm* involves the use of questions referring to the opinions of important people in general. It will involve five items with a seven point response format anchored by “strongly disagree…strongly agree”. Health professionals will report for example if they feel social pressure to perform the targeted behavior and if is expected from to perform the targeted behavior.

The measure of *perceived behavioral control* will involve four items with the same seven point response format. Items reflect people’s confidence that they are capable of performing the target behavior. Health professionals will report self-efficacy (e.g. is the targeted behavior difficult to perform) and their beliefs about the controllability of the behavior (e.g. whether I perform the behavior is completely up to me).

### Demographic characteristics of the health-professionals

We will collect data on sex, age, profession and years of working experience.

### Demographic and psychological characteristics of the knowledge broker

KBs will be asked about different themes that might influence their effectiveness to be a KB. These themes were identified from literature [14,23,24] and discussions amongst the project team. The following themes will be surveyed:

– Demographic data such as age and profession.

– How many years they work in the current organization

– The number of days of education in neurorehabilitation they followed in the past 3 years.

– Motivation to be a KB. Motivation will be assessed using a ten point scale.

– Generalized self-efficacy. This will be measured with the General Self Efficacy Scale-12 [22], a validated questionnaire on which subjects rate agreement with 12 items on 5-point scales ranging from “strongly agree” to “strongly disagree”.

### Result achieved

The results of the intervention as perceived by the KB will be measured using 3 items with a seven point response format anchored by “strongly disagree…strongly agree”. The items probed are if they are satisfied with the result, if they achieved their goal and if they think knowledge brokering resulted in more intensive therapy for stroke-patients.

### Organizational context

The KB will be asked how they perceive the conditions needed for health care improvement in their organization. This will be measured with seven items with a seven point response format anchored by “strongly disagree…strongly agree. The items probed are enough time (1 item), enough workforce (1 item) and enough sponsoring and experienced support from their management (4 items).

#### Semi structured interviews

Finally, 5 KBs will be asked to participate in semi-structured face to face interview to gather insights about their experiences with the brokering process and what they perceive to be of influence on their impact. The interview questions will be send to the KBs in advance so that they can prepare for the interview. All interviews will be recorded.

We will purposefully select the KBs that report to be the most and the least satisfied with the results they achieved.

### Data analyses

*Research question 1* To address this research question first of all, the support perceived by patients (4 questions) will be described by calculating the item frequencies at T0 and T1. Differences between T0 and T1 will be tested using the Mann–Whitney U test. Effect sizes will be calculated and the strength will be interpreted following the guidelines proposed by Cohen [25].

Secondly, we will analyze how many patients are ‘sufficiently supported’. To be considered ‘sufficiently supported’ a patient should perceive at least a little support on all 4 questions. For this purpose the 4 response-categories on these questions will be recoded in: 1 (no support, response category 1) and 2 (sufficiently supported, response-categories 2–4). A patient will be considered sufficiently supported if the score on all 4 questions is 2. The percentage of patients that are sufficiently supported at T0 and T1 will be calculated. The minimum guideline adherence we aim for in this study is that 50% of patients is sufficiently supported.

*Research question 2* For each health professional the targeted behavior will be determined by calculating the mean score on the 8 questions assessing the behavior and divided this by the number of patients cared for that day. The differences between T0 and T1 for this targeted behavior will be compared and significancy will be tested using paired T test. Effect sizes will be calculated and the strength will be interpreted following the guidelines proposed by Cohen [25].

Furthermore we will analyze how many health professionals sufficiently support their patients. A professional is considered to ‘sufficiently support’ patients if the targeted behavior-score is more than 0,5. The guideline adherence we aim for in this study is that more than 50% of health professionals sufficiently supports their patients.

*Research question 3* To test which determinants of the health professionals moderate change we will calculate a change score (post-pre) for the targeted behavior of health professionals. Correlations of these change scores with the TPB determinants and demographic factors of health professionals will be explored with spearmans rho.

The interrelationships among these determinants on the change in targeted behavior of health professionals will be further investigated using structural equation modelling.

*Research question 4* To test if characteristics of knowledge brokers and the organizational context moderate change for each stroke unit a mean change scores (post-pre) will be calculated for professional targeted behavior and patients’ perception.

At each stroke unit two or three KBs are active. For this analyses a mean KB score for the psychological and social demographic determinants will be calculated per stroke unit. Then correlations of the change scores with the psychological and demographic determinants of the KBs and the organizational context will be explored with spearmans rho. Multilevel analyses will be used to further analyze correlations.

Analyses will be performed using SPSS version PASW Statistics 20.0; alpha will be set at 0.05.

#### Interview-data

The interview-data will be analyzed qualitatively to further support or explain the quantitative outcomes.

#### Ethical approval

The Ethics Committee of the University Medical Center Utrecht has taken the position that this study does not fall within the remit of the Medical Research Involving Human Subjects Act since the participants are not subjected to procedures or required to follow certain rules of behavior. Written informed consent was thus not required.

### Progress of the study

22 rehabilitation centers and hospitals met the criteria and are participating in this study. They appointed in total 53 knowledge brokers. At T0, in June 2011, 783 questionnaires of health-professionals and 282 patient questionnaires were collected. At T1, in April 2012, 583 questionnaires of health-professionals and 238 patient questionnaires were collected. 316 health-professionals filled out a questionnaire at T0 and T1. The results are now being analyzed. The study will be completed in June 2014.

## Discussion

This paper presents the study design of a non-controlled pre-post study that will assess whether after the activities of knowledge brokers there is an increase in supportive behavior of health professionals to patients post stroke to exercise and be physically active. Furthermore it will assess the support patients perceive by health professionals to exercise and be physically active pre and post the activities of knowledge brokers.

We will be able to not only evaluate if desired changes were made, but also better understand if, why and when knowledge brokers seems to be beneficial and to identify determinants that predict which health professionals are susceptible to change their behavior. An innovative aspect of this study is the use a theoretical approach using the Theory of Planned Behavior to understand the factors that might modulate the effectiveness of knowledge brokers.

Strength of this study is its pragmatic approach [26]: we will investigate if the KB intervention works under usual conditions. The KBs will have a lot of freedom: they can choose what is sensible for them to implement and to tailor the intervention to local needs.

A weakness is the non-controlled pre-post study design. If any beneficial changes will be found, an experimental design will still be needed to make causal inferences on the effect of the KB intervention. Furthermore, we lack an objective measure of the supportive behavior of professionals. We will use self-reported measures and there is a risk that professionals overestimate their performance. An advantage of our study is however that next to self-reported measures also a patient reported outcome measure is assessed.

In summary, this study we will gain insight in when and why knowledge brokers seem to be effective. Also we will identify determinants that predict which health professionals are susceptible to change their behavior. This study will provide guidance how to implement guidelines and will help to improve stroke rehabilitation services.n summary this study will evaluate the use of KBs in rehabilitation centers and hospitals. This study will help understand how to implement guidelines, and improve stroke services. In the context of the growing number of patients that live with the longterm consequences of stroke, a large number of patients and health professionals may benefit from this intervention.

## Competing interests

The authors declare that they have no competing interests.

## Authors’ contributions

All authors contributed to the design and the protocol of the study. MW is the main researcher. All authors reviewed the manuscript and approved the final version.

## Pre-publication history

The pre-publication history for this paper can be accessed here:

http://www.biomedcentral.com/1472-6963/13/434/prepub
